# Assessment of Global and Regional Diffusion Changes along White Matter Tracts in Parkinsonian Disorders by MR Tractography

**DOI:** 10.1371/journal.pone.0066022

**Published:** 2013-06-13

**Authors:** Yulia Surova, Filip Szczepankiewicz, Jimmy Lätt, Markus Nilsson, Bengt Eriksson, Alexander Leemans, Oskar Hansson, Danielle van Westen, Christer Nilsson

**Affiliations:** 1 Department of Clinical Sciences, Neurology, Lund University, Lund, Sweden; 2 Department of Neurology Lund, Skåne University Hospital, Lund, Sweden; 3 Department of Medical Radiation Physics, Lund University, Lund, Sweden; 4 Center for Medical Imaging and Physiology, Skåne University Hospital, Lund, Sweden; 5 Lund University Bioimaging Center, Lund University, Lund, Sweden; 6 Clinical Memory Research Unit, Department of Clinical Sciences Malmö, Lund University, Sweden; 7 Image Sciences Institute, University Medical Center Utrecht, Utrecht, The Netherlands; 8 Department of Clinical Sciences, Diagnostic Radiology, Lund University, Lund, Sweden; University of Maryland, College Park, United States of America

## Abstract

**Purpose:**

The aim of the study was to determine the usefulness of diffusion tensor tractography (DTT) in parkinsonian disorders using a recently developed method for normalization of diffusion data and tract size along white matter tracts. Furthermore, the use of DTT in selected white matter tracts for differential diagnosis was assessed.

**Methods:**

We quantified global and regional diffusion parameters in major white matter tracts in patients with multiple system atrophy (MSA), progressive nuclear palsy (PSP), idiopathic Parkinson’s disease (IPD) and healthy controls). Diffusion tensor imaging data sets with whole brain coverage were acquired at 3 T using 48 diffusion encoding directions and a voxel size of 2×2×2 mm^3^. DTT of the corpus callosum (CC), cingulum (CG), corticospinal tract (CST) and middle cerebellar peduncles (MCP) was performed using multiple regions of interest. Regional evaluation comprised projection of fractional anisotropy (FA), mean diffusivity (MD), radial diffusivity (RD) and the apparent area coefficient (AAC) onto a calculated mean tract and extraction of their values along each structure.

**Results:**

There were significant changes of global DTT parameters in the CST (MSA and PSP), CC (PSP) and CG (PSP). Consistent tract-specific variations in DTT parameters could be seen along each tract in the different patient groups and controls. Regional analysis demonstrated significant changes in the anterior CC (MD, RD and FA), CST (MD) and CG (AAC) of patients with PSP compared to controls. Increased MD in CC and CST, as well as decreased AAC in CG, was correlated with a diagnosis of PSP compared to IPD.

**Conclusions:**

DTT can be used for demonstrating disease-specific regional white matter changes in parkinsonian disorders. The anterior portion of the CC was identified as a promising region for detection of neurodegenerative changes in patients with PSP, as well as for differential diagnosis between PSP and IPD.

## Introduction

Idiopathic Parkinson’s disease (IPD), progressive supranuclear palsy (PSP) and multiple system atrophy (MSA), are the most common neurodegenerative disease entities in what is often called parkinsonian disorders. Outside specialized centers and in the early stages of the diseases, clinical differential diagnosis can often be difficult because of similarity of symptoms and lack of diagnostic markers. Several imaging methods have been shown to be of benefit in the differential diagnosis of different parkinsonian disorders [1]. Diffusion tensor imaging (DTI) [2]–[3] with calculation of the fractional anisotropy (FA) and mean diffusivity (MD) have been used in the diagnostic evaluation of IPD, PSP and MSA [4]–[10]. Measurement of MD in basal ganglia structures can differentiate between IPD and MSA/PSP, while FA and MD values within specific white matter tracts can be helpful in differentiating PSP and the parkinsonian variant of MSA (MSA-P) from both each other and from IPD [11]–[19]. Few studies have been performed using diffusion tensor tractography (DTT) [20] in parkinsonian disorders. In a pilot study, we have previously shown that disease-specific degenerative changes can be demonstrated by DTT in MSA and PSP [4] and some of these findings have recently been confirmed [21]–[22]. However, global measurements of diffusion parameters in whole white matter tracts might overlook regional changes along a tract [23]–[24].

The aim of the present study was to investigate diffusion properties in major white matter tracts of patients with different parkinsonian disorders, employing DTT with an alternative processing scheme to be able to investigate both global and regional changes in larger nerve tracts [25]–[32]. We focused on three conventional parameters: FA, MD and radial diffusivity (RD) as well as a new measure of tract cross-sectional surface area - the apparent area coefficient (AAC) [30]. We demonstrate both tract-specific and disease-specific variations in DTT parameters along white matter tracts, which might form a basis for future studies of differential diagnosis and disease monitoring in parkinsonian disorders.

## Materials and Methods

### Ethics Statement

The Ethics Committee of Lund University approved this study. All study participants gave written consent for participation in the study, which was performed in accordance with the provisions of the Helsinki Declaration.

### Subjects

The study included 54 subjects: thirty-eight patients presenting parkinsonian syndromes and sixteen healthy controls. Patients were recruited from the Neurology and Memory Clinics at Skåne University Hospital and Landskrona Hospital, Sweden. Patients with a clinical diagnosis of probable IPD (n = 10), PSP (n = 16) and MSA-P (n = 12) according to established criteria [33]–[35] were included in the study. Clinical diagnoses were made by two neurologists experienced in parkinsonian disorders (C.N. and B.E). Out of the 16 patients with a diagnosis of probable PSP, all presented gradually progressive disorders with an age of onset 40 years or older, symmetry of symptoms (rigidity, bradykinesia); all patients presented both gaze palsy and prominent postural instability with falls within the first year of disease onset, and no response to dopaminergic drugs. All patients with MSA-P showed progressive akinesia and rigidity, urinary incontinence or incomplete bladder emptying after 1 year of disease onset as well as orthostatic hypotension, with no patients showing falls or gaze palsy in the first year of the disease. All patients with IPD showed good clinical improvement after administration of levodopa in respect to baseline conditions. Healthy controls matched for age and gender were recruited from the Swedish population registry. All healthy controls had a normal neurological examination and structural brain MRI, with no history of neurological or psychiatric disease.

### Data Acquisition

A 3 T Philips MR scanner, equipped with an eight-channel head coil, was used for the study. DTI was performed using a single-shot EPI sequence with diffusion encoding in 48 directions (*b* values 0 and 800 s/mm^2^) [36]. A b-value of 800 s/mm^2^ was selected to shorten the acquisition times. While the most commonly used b-value is 1000 s/mm^2^, DTI is expected to work well with b-values at least in the range b = 700–1200 s/mm^2^. However, lower b-values are expected to result in slightly higher values of the mean diffusivity [37]. The reconstructed voxel size was 2×2×2 mm^3^, and 60 slices were acquired. In order to shorten acquisition time and reduce susceptibility distortions, a SENSE factor of 2.5 was applied in the phase-encoding direction (anterior–posterior). The acquisition time for the DTI sequence was 6 min 49 s. The axial slices in the DTI volume were aligned with the posterior outline of the cranial brain stem.

### Data Processing and Fibre Tracking

Subject motion and eddy-current correction was performed in Elastix [38], as implemented in ExploreDTI [28], taking the b-matrix reorientation into account [39]. Whole-brain tractography was generated using ExploreDTI [28], with FA and angular threshold values of 0.2 and 30°, respectively. Multiple regions-of-interest (ROIs) were delineated on the directionally color-coded FA images, in order to extract three bilateral fibre structures: the middle cerebellar peduncle (MCP), the cingulum (CG), and the corticospinal tract (CST). In addition, the mid-sagittal segment (14 mm) of the corpus callosum (CC) was extracted. In addition to these four structures, the SCP and the ICP were also identified with the help of published DTI brain atlases [40]–[41]. However, the variability in tractography outcome of SCP and ICP was too high to permit any reliable analysis. As such, these structures were not considered for further investigation in this study.

Tractography of the frontal and parietal cingulum (CG) on each side was performed using colour-coded FA-maps. First, two ROIs were placed in the transversal plane to select the anterior part of the CG, which runs parallel to the genu of the corpus callosum; the most rostral part was not included. Then four ROIs were placed in the coronal plane at equal intervals along the superior part of the CG. Finally, two ROIs were placed in the transversal plane defining the posterior CG where it arches around the splenium of the CC. The descending part of the CG was not included. The data for CG were excluded in two patients with MSA-P and one patient with PSP due to incomplete tracking.

For the CST, we extracted the supratentorial portion of CST only, in order to limit variability caused by tracking over long distances [32]. For this purpose, three ROIs used as AND-gates in the tractography were placed in the axial plane, including the posterior limb of the internal capsule, centrum semiovale and the ipsilateral precentral gyrus (primary motor cortex), respectively. The CC was manually subdivided into five areas, according to Hofer’s scheme [42], although we treated CC3 and CC4 as one segment in the analysis. The anterior part of CC was defined as CC1–CC2, posterior – CC3–CC5. For the MCP two ROIs were placed: at the level of the pontine crossing fibres and at the level of the deep nuclei.

**Figure 1:**
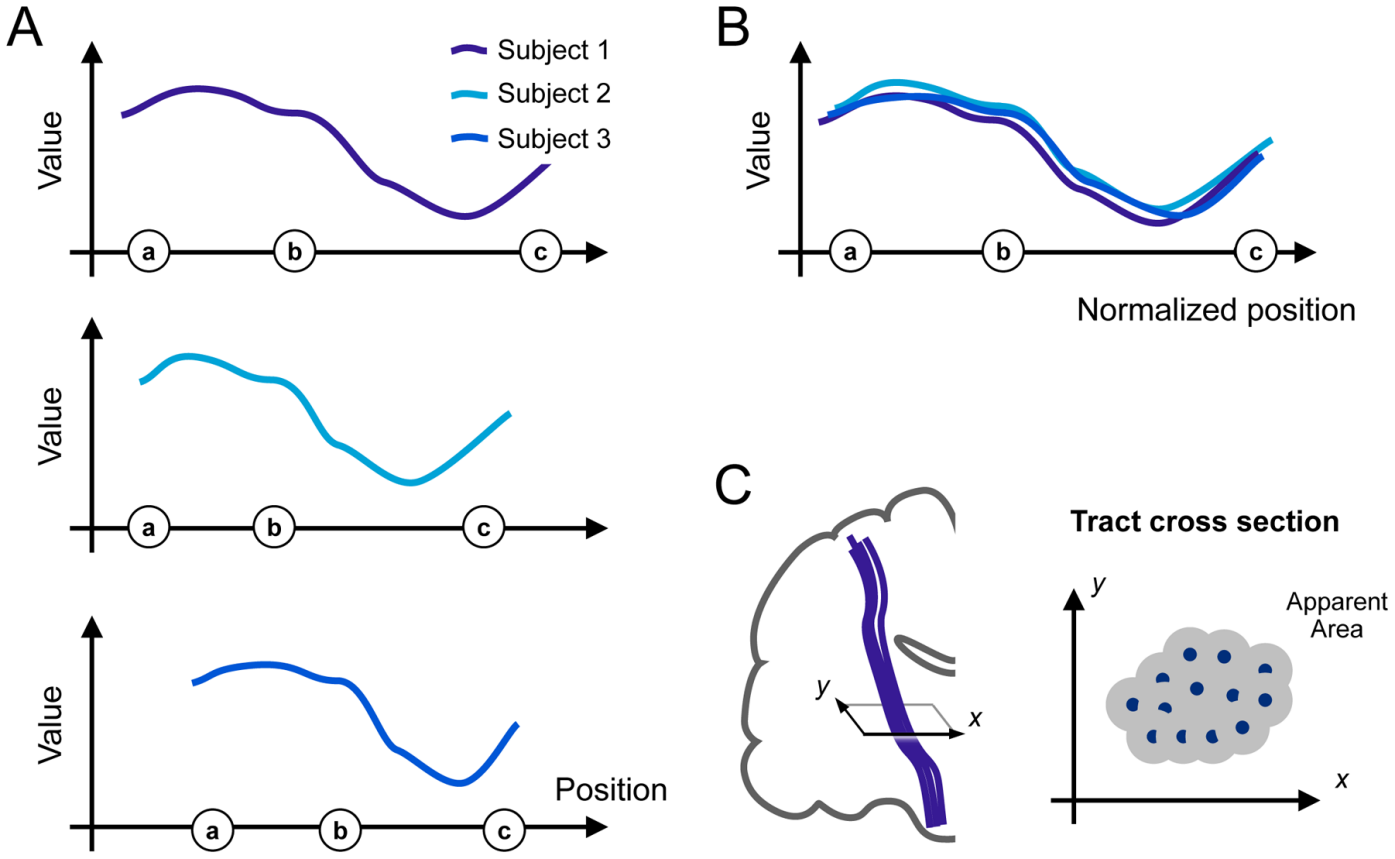
Schematic illustration of the method used to display the variation of the diffusion parameters along the tracts. Projection of diffusion parameters to a mean track resulted in individual parameter-versus-position plots (A), which were associated to the positions of the landmarks, shown as letters a-b-c in circles along the x-axis. The positions were normalized by interpolating the data so that the interval lengths a–b and b–c became equal in all individuals (B). Finally, the apparent area coefficient (AAC) of the tracts was calculated from cross sections of the tracts, where each individual track point (C, blue points) contributed to the total area coefficient with a weight of πr^2^, with r = 0.5 mm. Only non-overlapping parts of the subareas contributed to the total area coefficient.

In order to assess the variation of FA, MD, and RD along the white matter structures, the parameters were projected onto a calculated mean tract, which is a single tract that resembles the major features of each individual white matter structure in 3D space. This enables the evaluation of diffusion parameters as a function of position along the tract. The normalization was based on the position of the explicit landmarks. The method has been used previously [28], [30]. In principle, the method and workflow corresponds to the framework presented by Colby et al. [24], although no explicit tract resampling was performed. In addition to the diffusion parameters, we also calculated the cross-sectional area of the tract as a function of position, here denoted the apparent area coefficient (AAC) [30]. The value of the AAC was calculated from the track points passing through cross-sections of the tract, as shown in Fig. 1. FA, MD, RD and AAC will be referred to as DTT parameters. The CC was not analyzed in regard to AAC due to geometric limitations in the quantification of the structure’s apparent area along the mean track. Since we wanted to evaluate variations along the CC in an anterior-posterior direction the mean track of the CC was constructed differently than in the other pathways, i.e. with an orientation perpendicular to the fibre orientation. Thereby, the AAC, defined as the cross-sectional area of the tract in a plane with a normal given by the direction of the mean track, was not defined.

The mean values of FA, MD, RD and AAC from the tracts of the right and left hemisphere were treated as independent variables. In the cases where significant differences were found they were then included into a binary logistic regression analysis. This procedure was used as parkinsonian disorders affect both hemispheres, often asymmetrically, although there may be a similar extent of atrophy bilaterally in later stages. DTT parameters for the different groups were plotted as a function of the position along the mean track, with the distances between the anatomical landmarks scaled according to their average relative distance.

### Statistical Analysis

Statistical analysis was performed with SPSS Statistics 20 for Windows (IBM Corporation, Somers, NY, USA). Differences between groups in demographic and clinical categorical variables were analyzed by Fisher’s Exact test. The Kruskall-Wallis test was used to compare average FA, MD, RD and AAC values in whole tracts between the PSP, MSA-P, IPD and control groups. Where significant differences were found, group comparisons were performed using the Mann–Whitney *U-*test. An adjustment for multiple comparisons between the 4 control/patient categories (i.e. 6 comparisons) was made, leading to an adjusted significance level of *P* = 0.008 using Bonferroni correction. The average median values for the DTI parameters from the corresponding whole tracts of both hemispheres were reported. For statistical evaluation of differences in regional diffusion data between diagnostic subgroups, Mann-Whitney *U*-test was performed for each point along each white matter tract. Comparisons were made between controls and the respective disease groups at a significance level of P<0.05. To further study the regional variation within the CC, MD and FA in the anterior and posterior parts of CC were compared. Bee swarm box plots were applied to display the data graphically.

To study the ability of DTT measurements to distinguish IPD from PSP, univariate binary logistic regression analysis was performed with five diffusion parameter values that were significantly different between PSP and IPD, based on the results of Mann-Whitney *U*-test. All of these five models of binary logistic regression analysis were adjusted for age and sex. The sensitivity, specificity and the optimal cutoff level of DTI values chosen by the models were calculated with receiver operator characteristic curve analysis (ROC), as a measure of the usefulness of DTT in selected tracts as a diagnostic tool for individual cases.

## Results

Demographic and clinical data of patients and controls are reported in Table 1. There were no significant differences in age, gender ratio or disease duration between the IPD, MSA-P and PSP groups.

**Table 1:** Demographic data and clinical diagnosis.

	CTR (n = 16)	IPD (n = 10)	MSA-P (n = 12)	PSP (n = 16)	p
Sex female:male	7∶9	4∶6	8∶4	9∶7	0.583^a^
Age (years)	67 (63–73)	68 (59–70)	63 (56–75)	68 (65–72)	0.385^b^
Disease duration(years)	−	4.5 (2.0–7.5)	3.0 (2.2–5.0)	3.5 (2.2–4.0)	0.273^b^

There were no significant differences in demographic data between the controls and the different disease groups. All values expressed as medians, values in parenthesis indicate 25–75 percentiles.

a P values refer to Fisher’s Exact test,

b P values refer to Kruskal-Wallis test, where controls were excluded from the group comparisons of disease duration. IPD, idiopathic Parkinson’s disease; PSP, progressive supranuclear palsy; MSA-P, multiple system atrophy, parkinsonian variant; CTR, healthy controls.

Analysis of global values of DTT parameters was made by comparing median values in whole white matter tracts for the different disease groups. The most prominent differences were detected in FA, MD and RD values in the CC in patients with PSP compared with both IPD and controls. In addition, comparing PSP patients to IPD showed a significantly lower AAC in the CG and an increase of MD in the CST. There were no significant differences between PSP and MSA-P patients. We also found significantly higher AAC in the CG of patients with IPD compared to controls. MSA patients showed a significantly higher RD in the CST compared to IPD. There was an increase in MD and decrease in AAC in the MCP in MSA compared to IPD patients, which did not reach statistical significance. The median values of diffusion data and AAC for the whole tracts are summarized in Table 2.

**Table 2:** DTT parameters in white matter tracts.

		Group			
Tract	Parameter	CTR	IPD	MSA-P	PSP
CG	FA	0.50 (0.49–0.51)	0.51 (0.48–0.54)	0.47 (0.43–0.50)	0.46 (0.44–0.50)
	MD	0.83 (0.82–0.83)	0.83 (0.80–0.88)	0.84 (0.82–0.90)	0.85 (0.82–0.89)
	RD	0.58 (0.56–0.59)	0.57 (0.53–0.61)	0.62 (0.56–0.65)	0.62 (0.58–0.66)
	AAC	2.61 (2.51–2.78)	2.96^c^ (2.72–3.04)	2.81 (2.36–2.99)	2.44^be^ (2.27–2.72)
CST	FA	0.54 (0.53–0.55)	0.54 (0.54–0.57)	0.53 (0.51–0.54)	0.55 (0.53–0.58)
	MD	0.79 (0.78–0.82)	0.79 (0.77–0.80)	0.82 (0.80–0.84)	0.84^abe^ (0.81–0.87)
	RD	0.52 (0.50–0.54)	0.52 (0.49–053)	0.55^d^ (0.53–0.57)	0.55 (0.51–0.58)
	AAC	3.92 (3.59–4.70)	3.84 (3.60–4.22)	4.07 (3.82–4.61)	4.07 (3.59–4.47)
MCP	FA	0.61 (0.59–0.63)	0.59 (0.58–0.60)	0.58 (0.54–0.63)	0.59 (0.58–0.61)
	MD	0.74 (0.70–0.76)	0.72 (0.71–0.80)	0.80 (0.73–0.85)	0.77 (0.74–0.79)
	RD	0.43 (0.41–0.47)	0.44 (0.43–0.50)	0.46 (0.44–0.56)	0.47 (0.43–0.50)
	AAC	5.07 (4.77–5.27)	5.52 (4.92–5.69)	4.67 (4.38–5.27)	4.81 (4.41–5.14)
CC	FA	0.63 (0.60–0.65)	0.65 (0.62–0.67)	0.62 (0.60–0.63)	0.56^ab^ (0.53–0.61)
	MD	1.04 (0.99–1.10)	1.02 (0.96–1.07)	1.06 (1.02–1.13)	1.15^abe^ (1.08–1.26)
	RD	0.61 (0.56–0.69)	0.58 (0.52–0.64)	0.63 (0.59–0.69)	0.73^ab^ (0.65–0.87)

Fractional anisotropy (FA), mean and radial diffusivity (MD, RD, 10?−3 mmˆ2/s) and apparent area coefficient (AAC) values in major white matter tracts. The medians of diffusion parameters are presented. For paired structures (CG, cingulum, CST, corticospinal tract, MCP, middle cerebellar peduncles), all values are estimated medians from the left and right tracts; values in parenthesis indicate 25–75 percentiles.

a CTR/PSP, P≤0.008;

b IPD/PSP, P≤0.002;

c CTR/IPD, P = 0,002;

d IPD/MSA, P = 0.007, Mann-Whitney *U* test.

e IPD/PSP, P<0.05, binary logistic regression analysis, age/sex adjusted. Abbreviations: IPD, idiopathic Parkinson’s disease; PSP, progressive supranuclear palsy; MSA-P, multiple system atrophy, parkinsonian variant; CTR, healthy controls.

**Figure 2:**
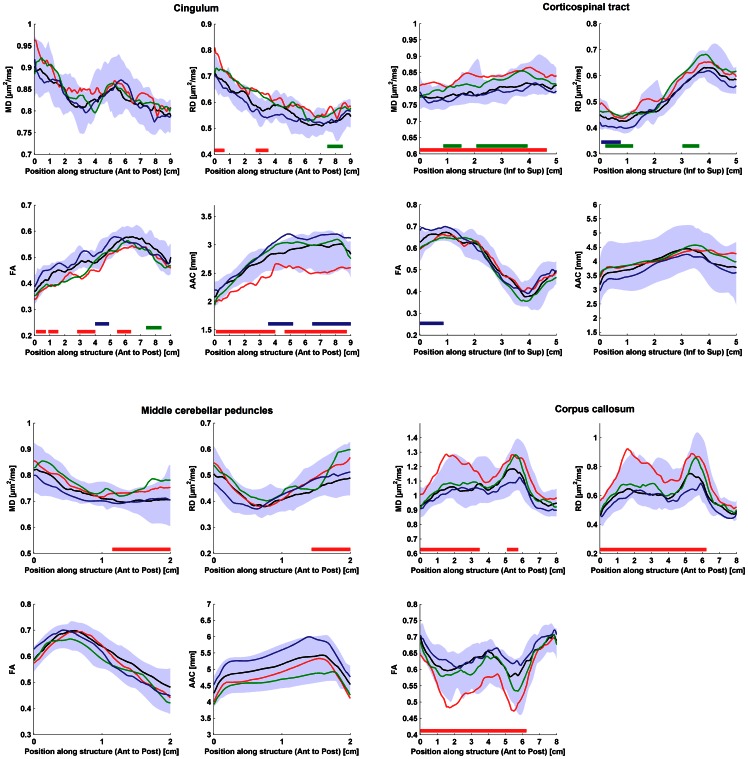
Comparisons between diffusion parameters and apparent area coefficient (AAC) in patients with Parkinsons’s disease (IPD – blue lines), multiple system atrophy (MSA – green lines), progressive supranuclear palsy (PSP – red lines) and controls (CTR – black lines). The lines show the median of diffusion parameters as a function of distance. The colored area shows the 10–90% confidence interval of the median in CTR. Panel A-C show mean diffusivity (MD), fractional anisotropy (FA), radial diffusivity (RD) and AAC in the cingulum, corticospinal tract, middle cerebellar peduncles and the corpus callosum, respectively. Values for AAC could not be calculated for the corpus callosum (see Methods). Positions with significant difference from controls (P<0.05, Mann–Whitney *U*-test) along tracts are marked with horizontal bars placed just above the x-axis, color-coded according to disease. Significant differences extending continuously for more than five mm along a tract were found for AAC in the cingulum, MD in the corticospinal tract, and RD, MD and FA in the corpus callosum in PSP.

**Figure 3:**
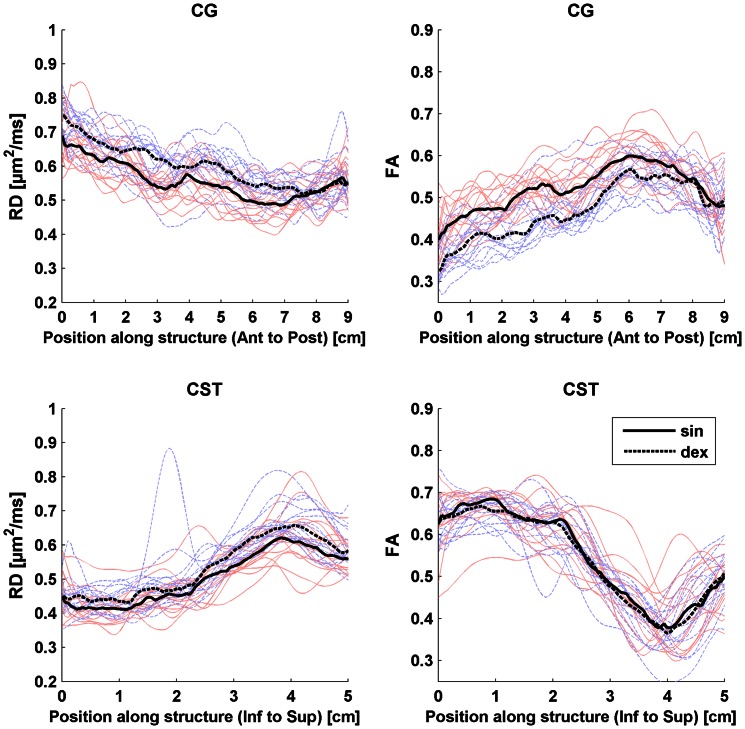
The plots show radial diffusivity (RD) and fractional anisotropy (FA) as functions of position along the cingulum (CG) (upper panels) and corticospinal tract (CST) (lower panels) from the right and left hemispheres in controls. Measurements from the right hemisphere are shown in blue and from the left side in red lines, respectively. The dashed and solid black lines represent the median value in each position of the left and right hand side tracts, respectively. The horizontal axis shows the position along CG in anterior – posterior direction. Distances 0–6 cm correspond approximately to the frontal part of the CG and 6–9 cm corresponds to the parietal part of the CG. For CST the horizontal axis shows the position along CST in inferior – superior direction. Distances 0–3 cm correspond approximately to the infracallosal part of the CST and 3–5 cm corresponds to the supracallosal part of the CST.

Concerning the regional analysis along white matter tracts, there were consistent variations of the DTT parameters along each tract, as determined by visual inspection, which were very similar in the different patient groups and controls. Each tract and parameter had its characteristic “2D-profile” along its length (Fig. 2). The exception was the diffusion values from CC in the PSP group that differed significantly in shape from the other groups (see below and Fig. 2). For all parameters, there were differences in FA and RD values between the left and right CG (Fig. 3) and, to a lesser degree, for MD in the CST (data not shown), which were consistent throughout the control and patient groups (Fig. 3). Although all statistically significant differences between controls and disease groups are depicted in Fig. 2, only continuous changes encompassing more than 2 cm along a tract were considered of significance for further analysis. There was a trend towards lower AAC in both the MSA and PSP groups in MCP, which was not significant (Fig. 2). In PSP, significant changes were seen for AAC in the CG and for MD in the CST. However, the most striking finding was a marked increase in MD and RD, and a corresponding reduction of FA, in the anterior and central parts of the CC in PSP.

Based on the Mann-Whitney *U*-test, five models of univariate binary logistic regression analysis were performed in order to test the potential of using diffusion parameters for differential diagnosis of IPD and PSP. AAC in the CG, MD in the CST, MD in the CC, RD in the CC and FA in the CC were included in the models, see Statistical analysis. A summary of the results is shown in Table 3, with details of each comparison given below. Logistic regression analysis confirmed that the AAC in the CG, the MD in CST and the MD in the CC could significantly (P<0.025) discriminate PSP from IPD (Table 3). The sensitivity and specificity for all these parameters, calculated using a ROC curve analysis, showed the optimal cutoff levels (with an area under the ROC curve of 0.85–0.88) to discriminate PSP from IPD with a sensitivity of 81–94% and a specificity of 80%. This correctly classified 80–87% of PSP and IPD subjects.

**Table 3:** Use of DTT parameters for differential diagnosis.

Structure	Parameter	AUC (ROC)	Cutoff	Sensitivity, %	Specificity, %	Observed clinical diagnosis	Predicted			
							Clinical diagnosis		Percentage correct diagnosis, %	Overall percentage correct diagnosis, %
							IPD	PSP		
CG Whole*	AAC	0,88	2,730	87	80	IPD	8	2	80	80
						PSP	3	12	80	
CST Whole*	MD	0,85	0,801	94	80	IPD	7	3	70	87
						PSP	3	13	81	
CC Whole*	MD	0,85	1,072	81	80	IPD	7	3	70	81
						PSP	2	14	87	

Mean diffusivity (MD, 10?−3 mmˆ2/s) and apparent area coefficient (AAC) differentiating PSP from IPD. CG – cingulum, CST – corticospinal tract, CC – corpus callosum, PSP – progressive supranuclear palsy, AUC – area under curve, ROC – receiver operating characteristic analysis.

* Significant differences between PSP and IPD, P<0,05, using binary logistic regression, adjusted for age and sex. There were no age/sex differences between IPD and PSP groups together with chosen MR parameters.

**Figure 4:**
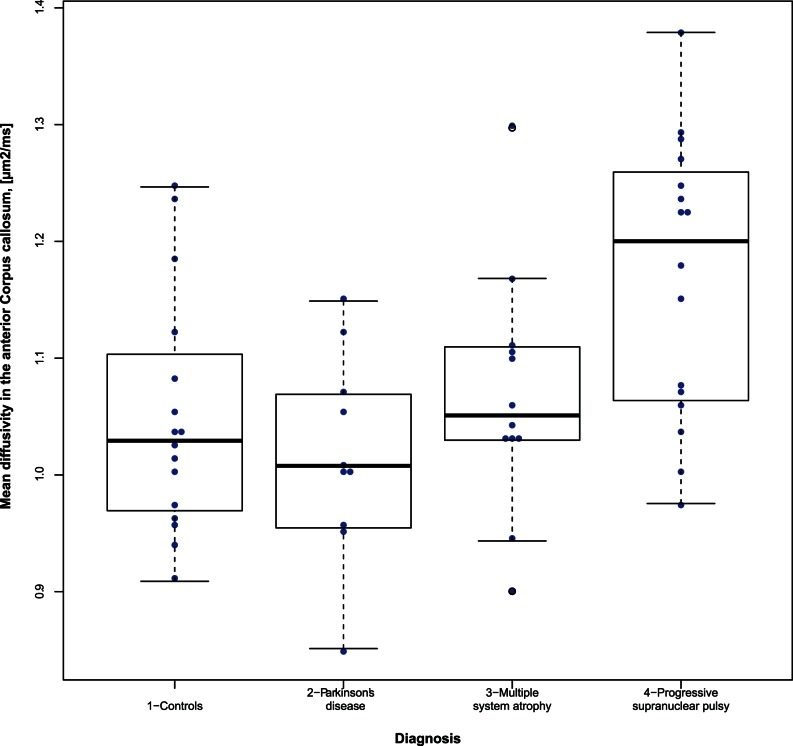
Beeswarm box-plot comparing mean diffusivity and fractional anisotropy in the anterior part of corpus callosum between patients with Parkinson’s disease, multiple system atrophy, progressive supranuclear palsy and controls. Horizontal lines that intersect the boxes are medians. The top of the boxes is the 75th percentile and the bottom the 25th percentile. The whiskers above and below boxes represent maximum and minimum values when there are no outliers. If outliers are present the whisker on the appropriate side is taken to 1.5×IQR from the quartile. Outliers are labeled with open circles.

To further evaluate the differences in regional values of diffusion parameters along white matter tracts demonstrated above, diffusion parameters from the anterior and posterior part of CC were compared separately. The MD in the anterior part of CC, tested in the model of binary logistic regression, age and sex adjusted, showed the same significant trend toward discrimination of PSP and IPD as the MD in the whole CC. MD in the posterior part of the CC did not reach significance in the binary logistic regression model. Bee swarm box plots showed substantial overlap between the different groups (Fig. 4). The controls also showed large variation of diffusion parameter values.

## Discussion

Diffusion tensor imaging has emerged as a powerful tool for detecting early degenerative changes in both normal aging [43]–[44] and neurodegenerative disease [45], [46]. Studies have shown that diffusion changes can be detected before atrophy or signal changes can be seen on standard MRI sequences [46]–[47]. It is therefore natural that DTI has been used to explore diffusion changes also in parkinsonian disorders [48]–[50].

The first study that demonstrated diffusion changes in the parkinsonian brain was Yoshikawa et al. [50], using a ROI-based approach. They showed that by placing small ROIs along the presumed position of the nigrostriatal tract, reduced ADC and increased FA could be seen in both patients with IPD and PSP compared to controls. DTI with manual placement of ROIs has since then dominated and has been applied to both subcortical nuclei and white matter tracts, as reviewed above. Although most studies have focused on group differences between controls and disease groups, there have been attempts to determine cut-off values for diffusion parameters in specific structures that can aid in the differential diagnosis between IPD and atypical parkinsonian disorders such as MSA and PSP. While measurements of FA and MD in the putamen in most cases can differentiate MSA and PSP from IPD and controls [12], [51], other studies have targeted the middle and superior cerebellar peduncles to differentiate between MSA and PSP [4], [13], [52].

Using DTI with a ROI-based approach is time-consuming and has the additional disadvantage of only capturing a small part of a white matter tract. With larger ROIs there is a risk that included voxels might contain signal from adjacent tissue including other tracts, nuclei or CSF, a problem which becomes even more pronounced when investigating smaller white matter tracts [53]. In addition, ROI-based approaches also result in loss of data on local variations in diffusion parameters [23]. To address this issue some studies have used tract-based spatial statistics (TBSS) for analysis of DTI-data between groups [54]–[55]. TBSS captures regional variations along white matter tracts which can be correlated to clinical symptoms in comparisons between groups [55], but does not allow for specific analysis of tracts in individual patients.

DTT allows for delineation and separate analysis of diffusion parameters and structure in discrete white matter tracts [24], [27], [28]. It has so far only been used in a limited number of studies in parkinsonian disorders [1], [4], [21], [23]. Although previous studies have demonstrated regional variations along normal white matter tracts as well as in neurodegenerative disease [23], [32], [55], most studies to date have used the mean value of diffusion parameters in the whole tract for analysis. In addition, a quantitative measure of tract size has largely been lacking.

In this study, we applied a new approach for visualization and quantitative evaluation of DTT parameters along white matter tracts in patients with parkinsonian disorders as a function of distance from specific anatomical landmarks [27], [28], [31]. The procedure overcomes existing limitations of user-specified region definition or full-brain registration. The tracking takes only a few seconds on a standard PC and the whole process including ROI specifications, DTT parameter calculation, tracking, and storing fibres into a database takes a few minutes per subject. Our results demonstrate that the method is highly reproducible and captures known variations along specific white matter tracts. The difference between the right and left cingulum shown here has previously been demonstrated in healthy adults [56], as has variations along the corticospinal tract [32]. It is interesting that these variations change or disappear in PSP where the disease process often involves both these pathways. The numerical values for FA and MD in the whole tracts showed good agreement with published data from normal individuals in the age group 40–65 years for CG, CST and MCP [57]. Our present method of analysis seems most suitable for analysis of larger white matter tracts. Diffusion data from tractography of the SCP and ICP resulted in very large variations in diffusion parameters precluding any further statistical analysis. Current research in our group addresses the issues of developing more advanced schemes for fibre tract alignment and validation of fibre bundles obtained by tracking in comparison to co-registered structural image data.

There are results from other studies, suggesting that the MCP has the best diagnostic accuracy in discriminating MSA from IPD and PSP [13]. Regional analysis in our study showed that DTT parameters appeared to be changed along the entire or larger part of the MCP (MD, RD and AAC) in MSA-P, compared to controls, although the changes were non-significant. In fact, significant differences were only found for the posterior MCP in patients with PSP. Degeneration of the olivopontocerebellar system, including the pontine neurons and transverse fibres, is highly variable and mostly less severe in MSA-P as compared to the cerebellar form of MSA (MSA-C), which can explain the variation and overlap with controls and other disease groups [58]–[60]. Our results to a certain degree support that the pathological process affects the pons and cerebellum also in MSA-P, but that the discriminatory potential for differential diagnosis is limited.

The current study using DTT demonstrates that the CC is the structure that best differentiates PSP from IPD and MSA, as has previously been found in studies using DTI [6], [7], [12], [21], [54]. In addition, our study indicates that the most prominent changes in diffusion parameters compared to other parkinsonian disorders occur in the anterior part of CC in PSP, in keeping with the well-known involvement of the frontal lobes in this disease [54], [61], [62]. Interestingly, reduced FA values in the genu of CC has recently been reported in patients with Parkinson’s disease with dementia (PDD) and dementia with Lewy bodies (DLB) as well [63]–[64]. In addition, Kamagata et al. demonstrated reduced FA in the anterior CG of patients with PD [65], a finding which was not reproduced in the present study.

We also found that MD values in the CST can help to discriminate IPD and PSP. It is well established that CST can be affected in PSP [4]. The use of AAC as a measure of tract diameter appears to be a useful addition to DTT. Although this study was mainly exploratory (hypothesis-generating), comparing diffusion parameters at group level, we have also demonstrated significant discriminatory power for differentiation of IPD and PSP using AAC in CG as well as MD in CST and CC. These results should be treated with caution considering the limited number of cases in each group, but may add useful information for future studies.

Measurement of DTT parameters in whole white matter tracts was sufficient for detecting clinically important differences between patient groups, while regional analysis of diffusion data helped to determine the location of the changes. Our results highlight the need to consider differences in diffusion properties along major white matter tracts and the possibility of asymmetry between the left and right hemispheres both in patients and healthy controls. The large degree of overlap in diffusion parameter values between individuals limits the usefulness of the method as a diagnostic biomarker on its own. However, DTT parameters could still be used together with other clinical, biochemical and imaging markers for diagnostic purposes. Large inter-individual differences in clinical signs and biomarker values are common in neurodegenerative disease as well as in other biological systems. For this reason even extreme outliers were included in the analysis. It is important to note the large variation in diffusion parameter values also in neurologically healthy elderly persons.

Several limitations of our study should be mentioned. First, the relatively small number of patients limits generalization of the findings. Moreover, positioning of the ROIs was performed manually and errors could be introduced by limited intrarater reliability. Also, statistical analysis within small ROIs including only a few voxels might be very sensitive to partial volume effects, limiting the method to the study of larger tracts. Although all patients fulfilled clinical research criteria for diagnosis of their respective disorder, they might still vary substantially in disease stage, rate of progression and clinical symptoms. Additionally, none of the included subjects had their diagnosis confirmed by autopsy. However, diagnosis of MSA and PSP by an experienced clinician yields sensitivities of 88% and 84% (positive predictive values of a clinical diagnosis of MSA and PSP in this study were 86 and 80%, respectively) [66]. Finally, we acknowledge that DTI is not capable of unambiguously characterizing the white matter microstructure in regions of complex fibre architecture [67]–[68] and that more advanced diffusion approaches could be preferable for reconstructing tract pathways [69]–[71]. Notwithstanding the low specificity in assessing the cause of the observed diffusion abnormalities [72], DTI may still exhibit a high sensitivity, which has shown to be useful in several applications [73]–[74].

In summary, we have shown that DTT has the potential as a tool for assessing pathway-specific abnormalities in parkinsonian disorders on both an individual and group level. The ability to visualize and quantify global and regional DTT parameters in specific white matter tracts could improve differential diagnosis and also help to explain the underlying anatomical mechanisms of individual clinical phenotypes. Additional studies are required to validate the research findings and to determine whether DTI/DTT can detect diffusion changes in very early stages of parkinsonian disorders.

## References

[1] Piccini P, Brooks DJ (2006) New developments of brain imaging for Parkinson’s disease and related disorders. Mov Disord 21: 2035–41. 16874751 10.1002/mds.20845

[2] Basser PJ, Mattiello J, LeBihan D (1994) MR Diffusion Tensor Spectroscopy and Imaging. Biophysical Journal 66: 259–67. 8130344 10.1016/S0006-3495(94)80775-1 PMC1275686

[3] Tournier JD, Mori S, Leemans A (2011) Diffusion Tensor Imaging and Beyond. Magn Reson Med 65: 1532–56. 21469191 10.1002/mrm.22924 PMC3366862

[4] Nilsson C, Markenroth Bloch K, Brockstedt S, Latt J, Widner H, et al (2007) Tracking the neurodegeneration of parkinsonian disorders – a pilot study. Neuroradiology 49: 111–19. 17200869 10.1007/s00234-006-0165-1

[5] Seppi K, Schocke MF, Mair KJ, Esterhammer R, Scherfler C, et al (2006) Progression of putaminal degeneration in multiple system atrophy: a serial diffusion MR study. Neuroimage 31: 240–45. 16443375 10.1016/j.neuroimage.2005.12.006

[6] Wang J, Wai Y, Lin WY, Ng S, Wang CH, et al (2010) Microstructural changes in patients with progressive supranuclear palsy: a diffusion tensor imaging study. J Magn Reson Imaging 32: 69–75. 20578012 10.1002/jmri.22229

[7] Padovani A, Borroni B, Brambati SM, Agosti C, Broli M, et al (2006) Diffusion tensor imaging and voxel based morphometry study in early progressive supranuclear palsy. J Neurol Neurosurg Psychiatry 77: 457–63. 16306152 10.1136/jnnp.2005.075713 PMC2077489

[8] Peran P, Cherubini A, Assogna F, Piras F, Quattrocchi C, et al (2010) Magnetic resonance imaging markers of Parkinson’s disease nigrostriatal signature. Brain 133: 3423–33. 20736190 10.1093/brain/awq212

[9] Zhan W, Kang GA, Glass GA, Zhang Y, Shirley C, et al (2012) Regional alterations of brain microstructure in Parkinson’s diseases using diffusion tensor imaging. Mov Disord 1: 90–97. 10.1002/mds.23917 PMC4472452 21850668

[10] Vaillancourt DE, Spraker MB, Prodoehl J, Abraham I, Corcos DM, et al (2009) High-resolution diffusion tensor imaging in the substantia nigra of de novo Parkinson disease. Neurology 72: 1378–84. 19129507 10.1212/01.wnl.0000340982.01727.6e PMC2677508

[11] Nicoletti G, Tonon C, Lodi R, Condino F, Manners D, et al (2008) Apparent diffusion coefficient of the superior cerebellar peduncle differentiates progressive supranuclear palsy from Parkinson’s disease. Mov Disord 23: 2370–76. 18816803 10.1002/mds.22279

[12] Kollensperger M, Seppi K, Liener C, Boesch S, Heute D, et al (2007) Diffusion weighted imaging best discriminates PD from MSA-P: A comparison with tilt table testing and heart MIBG scintigraphy. Mov Disord 22: 1771–76. 17579357 10.1002/mds.21614

[13] Paviour DC, Thornton JS, Lees AJ, Jäger HR (2007) Diffusion-weighted magnetic resonance imaging diffentiates parkinsonian variant of multiple-system atrophy from progressive supranuclear palsy. Mov Disord 22: 68–74. 17089396 10.1002/mds.21204

[14] Erbetta A, Mandelli ML, Savoiardo M, Grisoli M, Bizzi A, et al (2009) Diffusion tensor imaging shows different topographic involvement of the thalamus in progressive supranuclear palsy and corticobasal degeneration. Am J Neuroradiol 30: 1482–87. 19589886 10.3174/ajnr.A1615 PMC7051630

[15] Ito S, Makino T, Shirai W, Hattori T (2008) Diffusion tensor analysis of corpus callosum in progressive supranuclear palsy. Neuroradiology 50: 981–85. 18779957 10.1007/s00234-008-0447-x

[16] Tha KK, Terae S, Yabe I, Miyamoto T, Soma H, et al (2010) Microstructural white matter abnormalities of multiple system atrophy: in vivo topographic illustration by using diffusion-tensor MR imaging. Radiology 255: 563–9. 20413765 10.1148/radiol.10090988

[17] Shiga K, Yamada K, Yoshikawa K, Mizuno T, Nishimura T, et al (2005) Local tissue anisotropy decreases in cerebellopetal fibres and pyramidal tract in multiple system atrophy. J Neurol 252: 589–96. 15834652 10.1007/s00415-005-0708-0

[18] Schocke MF, Seppi K, Esterhammer R, Kremser C, Mair KJ, et al (2004) Trace of diffusion tensor differentiates the Parkinson variant of multiple system atrophy and Parkinson’s disease. Neuroimage 21: 1443–51. 15050569 10.1016/j.neuroimage.2003.12.005

[19] Schocke MF, Seppi K, Esterhammer R, Kremser C, Jaschke W, et al (2002) Diffusion-weighted MRI differentiates the Parkinson variant of multiple system atrophy from PD. Neurology 58: 575–80. 11865135 10.1212/wnl.58.4.575

[20] Mori S, Crain BJ, Chacko VP, van Zijl PC (1999) Three-dimensional tracking of axonal projections in the brain by magnetic resonance imaging. Ann Neurol 45: 265–9. 9989633 10.1002/1531-8249(199902)45:2<265::aid-ana21>3.0.co;2-3

[21] Canu E, Agosta F, Baglio F, Galantucci S, Nemni R, et al (2011) Diffusion tensor magnetic resonance imaging tractography in progressive supranuclear palsy. Mov Disord 26: 1751–5. 10.1002/mds.23739 21500281

[22] Makino T, Ito S, Kumabara S (2011) Involvement of pontine transverse and longitudinal fibers in multiple system atrophy: a tractography-based study. J Neurol Sci 303: 61–6. 21310434 10.1016/j.jns.2011.01.014

[23] Kvickström P, Eriksson B, van Westen D, Lätt J, Elfgren C, et al (2011) Selective frontal neurodegeneration of the inferior fronto-occipital fasciculus in progressive supranuclear palsy (PSP) demonstrated by diffusion tensor tractography. BMC Neurology. 26: 11–13. 10.1186/1471-2377-11-13 PMC3041656 21269463

[24] Colby JB, Soderberg L, Lebel C, Dinov ID, Thompson PM, et al (2012) Along-tract statistics allow for enhanced tractography analysis. Neuroimage 59: 3227–42. 22094644 10.1016/j.neuroimage.2011.11.004 PMC3288584

[25] Lim KO, Hedehus M, Moseley M, de Crespiqny A, Sullivan EV, et al (1999) Compromised white matter tract integrity in schizophrenia inferred from diffusion tensor imaging. Arch Gen Psychiatry 56: 367–374. 10197834 10.1001/archpsyc.56.4.367

[26] Xu D, Mori S, Solaiyappan M, van Zijl PC, Davatzikos C (2002) A framework for callosal fiber distribution analysis. Neuroimage 17: 1131–43. 12414255 10.1006/nimg.2002.1285

[27] Fillard P, Gilmore J, Piven J, Lin WL, Gerig G (2003) Quantitative analysis of white matter fiber properties along geodesic paths. In: Ellis RE, Peters TM, editors. Medical Image Computing and Computer-Assisted Intervention (MICCAI 03). Lecture Notes in Computer Science, Volume 2879. Heidelberg: Springer-Verlag. 16–23.

[28] Leemans A, Jeurissen B, Sijbers J, Jones DK (2009) ExploreDTI: a graphical toolbox for processing, analyzing, and visualizing diffusion MR data. In Proc Intl Soc Mag Reson Med 3537.

[29] Szczepankiewicz F, Leemans A, Sundgren P, Wirestam R, Ståhlberg F, et al.. (2012) Power and variability analysis in diffusion kurtosis imaging: Sample size estimation in three white matter structures. In Proc Intl Soc Mag Reson Med 3628.

[30] Mårtensson J, Nilsson M, Ståhlberg F, Sundgren P, Nilsson C, et al.. (2013) Spatial analysis of diffusion tensor tractography statistics along the inferior fronto-occipital fasciculus with application in progressive supranuclear palsy. MAGMA (In press). 10.1007/s10334-013-0368-5 23543132

[31] Mårtensson J, Nilsson M, Elfgren C, Landqvist M, Ståhlberg F, et al.. (2011) Spatial analysis of diffusion tensor tractography depicts local white matter changes. In Proc Intl Soc Mag Reson Med 4876.

[32] Reich DS, Smith SA, Jones CK, Zackowski KM, van Zijl PC, et al (2006) Quantitative Characterization of the Corticospinal Tract at 3 Tesla. Am J Neuroradiol 27: 2168–78. 17110689 PMC2802715

[33] Calne DB, Snow BJ, Lee C (1992) Criteria for diagnosing Parkinson’s disease. Ann Neurol 32: S125–7. 1510370 10.1002/ana.410320721

[34] Litvan I, Agid Y, Calne D, Campbell G, Dubois B, et al (1996) Clinical research criteria for the diagnosis of progressive supranuclear palsy (Steele-Richardson-Olszewski syndrome): report of the NINDS-SPSP International Workshop. Neurology 47: 1–9. 8710059 10.1212/wnl.47.1.1

[35] Gilman S, Low PA, Quinn N, Albanese A, Ben-Shlomo Y, et al (1999) Consensus statement on the diagnosis of multiple system atrophy. J Neurol Sci 163: 94–8. 10223419 10.1016/s0022-510x(98)00304-9

[36] Jones DK, Leemans A (2010) Diffusion tensor imaging. Methods Mol Biol 711: 127–44. 10.1007/978-1-61737-992-5_6 21279600

[37] Nucifora PG, Verma R, Lee SK, Melhem ER (2007) Diffusion-tensor MR imaging and tractography: exploring brain microstructure and connectivity. Radiology 245: 127–44. 10.1148/radiol.2452060445 17940300

[38] Klein S, Staring M, Murphy K, Viergever MA, Pluim JP (2010) Elastix: A toolbox for intensitybased medical image registration. IEEET. Med Imaging 29: 195–205. 10.1109/TMI.2009.2035616 19923044

[39] Leemans A, Jones DK (2009) The B-matrix must be rotated when correcting for subject motion in DTI data. Magn Reson Med 61: 1336–49. 19319973 10.1002/mrm.21890

[40] Stieltjes B, Kaufmann WE, van Zijl PC, Fredericksen K, Pearlson GD, et al (2001) Diffusion tensor imaging and axonal tracking in the human brainstem. Neuroimage 14: 723–35. 11506544 10.1006/nimg.2001.0861

[41] Wakana S, Jiang H, Nagae-Poetscher LM, van Zijl PC, Mori S (2004) Fiber tract-based atlas of human white matter anatomy. Radiology 230: 77–87. 14645885 10.1148/radiol.2301021640

[42] Hofer S, Frahma J (2006) Topography of the human corpus callosum revisited: comprehensive fiber tractography using diffusion tensor magnetic resonance imaging. Neuroimage 32: 989–94. 16854598 10.1016/j.neuroimage.2006.05.044

[43] Hsu JL, Van Hecke W, Bai CH, Lee CH, Tsai YF, et al (2010) Microstructural white matter changes in normal aging: a diffusion tensor imaging study with higher-order polynomial regression models. Neuroimage 49: 32–43. 19699804 10.1016/j.neuroimage.2009.08.031

[44] Hsu JL, Leemans A, Bai CH, Lee CH, Tsai YF, et al (2008) Gender differences and age-related white matter changes of the human brain: a diffusion tensor imaging study. Neuroimage 39: 566–77. 17951075 10.1016/j.neuroimage.2007.09.017

[45] Zhang Y, Schuff N, Jahng GH, Bayne W, Mori S, et al (2007) Diffusion tensor imaging of cingulum fibers in mild cognitive impairment and Alzheimer disease. Neurology 68: 13–9. 17200485 10.1212/01.wnl.0000250326.77323.01 PMC1941719

[46] Muller MJ, Greverus D, Weibrich C, Dellani PR, Scheurich A, et al (2007) Diagnostic utility of hippocampal size and mean diffusivity in amnestic MCI. Neurobiol Aging 28: 398–403. 16529847 10.1016/j.neurobiolaging.2006.01.009

[47] Salat DH, Lee SY, Yu P, Setty B, Rosas HD, et al.. (2009) DTI in development and aging. In: Johansen-Berg H and Behrens TEJ (eds) Diffusion MRI. From quantitative measurement to in vivo neuroanatomy. Amsterdam, Elsevier/Academic Press. 205–36.

[48] Van Camp N, Blockx I, Verhoye M, Casteels C, Coun F, et al (2009) Diffusion tensor imaging in a rat model of Parkinson's disease after lesioning of the nigrostriatal tract. NMR Biomed 22: 697–706. 19378292 10.1002/nbm.1381

[49] Wang HC, Hsu JL, Leemans A (2012) Diffusion Tensor Imaging of Vascular Parkinsonism: Structural Changes in Cerebral White Matter and the Association With Clinical Severity. Arch Neurol 23: 1–9. 10.1001/archneurol.2012.633 22825428

[50] Yoshikawa K, Nakata Y, Yamada K, Nakagawa M (2004) Early pathological changes in the parkinsonian brain demonstrated by diffusion tensor. Neurol Neurosurg Psychiatry 75: 481–4. 10.1136/jnnp.2003.021873 PMC1738942 14966170

[51] Seppi K, Schocke MF, Esterhammer R, Kremser C, Brenneis C, et al (2003) Diffusion-weighted imaging discriminates progressive supranuclear palsy from PD, but not from the parkinson variant of multiple system atrophy. Neurology 60: 922–7. 12654954 10.1212/01.wnl.0000049911.91657.9d

[52] Tsukamoto K, Matsusue E, Kanasaki Y, Kakite S, Fujii S, et al (2012) Significance of apparent diffusion coefficient measurement for the differential diagnosis of multiple system atrophy, progressive supranuclear palsy, and Parkinson's disease: evaluation by 3.0 T MR imaging. Neuroradiology 54: 947–55. 22274571 10.1007/s00234-012-1009-9

[53] Vos SB, Jones DK, Viergever MA, Leemans A (2011) Partial volume effect as a hidden covariate in DTI analyses. Neuroimage 55: 1566–76. 21262366 10.1016/j.neuroimage.2011.01.048

[54] Knake S, Belke M, Menzler K, Pilatus U, Eqqert KM, et al (2010) In Vivo Demonstration of Microstructural Brain Pathology in Progressive Supranuclear Palsy: A DTI Study Using TBSS. Mov Disord 25: 1232–8. 20222139 10.1002/mds.23054

[55] Whitwell JL, Master AV, Avula R, Kantarci K, Eqqers SD, et al (2011) Clinical correlates of white matter tract degeneration in progressive supranuclear palsy. Arch Neurol 68: 753–60. 21670399 10.1001/archneurol.2011.107 PMC3401587

[56] Gong G, Jiang T, Zhu C, Zang Y, Wanq F, et al (2005) Asymmetry analysis of cingulum based on scale-invariant parameterization by diffusion tensor imaging. Hum Brain Mapp 24: 92–8. 15455461 10.1002/hbm.20072 PMC6871701

[57] Lee CEC, Danielian LE, Thomasson D, Baker EH (2009) Normal regional fractional anisotropy and diffusion coefficient of the brain measured on a 3TMR scanner. Neuroradiology 51: 3–9. 18704391 10.1007/s00234-008-0441-3

[58] Kume A, Takahashi A, Hashizume Y (1993) Neuronal cell loss of the striatonigral system in multiple system atrophy. J Neurol Sci 117: 33–40. 8410064 10.1016/0022-510x(93)90151-n

[59] Wenning GK, Tison F, Ben Shlomo Y, Daniel SE, Quinn NP (1997) Multiple system atrophy: a review of 203 pathologically proven cases. Mov Disord 12: 133–47. 9087971 10.1002/mds.870120203

[60] Köllensperger M, Geser F, Ndayisaba JP, Boesch S, Seppi K, et al (2010) Presentation, diagnosis and management of multiple system atrophy in Europe: final analysis of the European multiple system atrophy registry. Mov Disord 25: 2604–12. 20922810 10.1002/mds.23192

[61] Brenneis C, Seppi K, Schocke M, Benke T, Wenning GK, et al (2004) Voxel based morphometry reveals a distinct pattern of frontal atrophy in progressive supranuclear palsy. J Neurol Neurosurg Psychiatry 75: 246–9. 14742598 PMC1738933

[62] Cordato NJ, Duggins AJ, Halliday GM, Morris JG, Pantelis C (2005) Clinical deficits correlate with regional cerebral atrophy in progressive supranuclear palsy. Brain 128: 1259. 15843423 10.1093/brain/awh508

[63] Kamagata K, Motoi Y, Tomiyama H, Abe O, Ito K, et al.. (2013) Relationship between cognitive impairment and white-matter alteration in Parkinson's disease with dementia: tract-based spatial statistics and tract-specific analysis. Eur Radiol 13: In press. 10.1007/s00330-013-2775-4 PMC3674338 23404139

[64] Hattori T, Orimo S, Aoki S, Ito K, Abe O, et al (2012) Cognitive status correlates with white matter alteration in Parkinson's disease. Hum Brain Mapp 33: 727–39. 21495116 10.1002/hbm.21245 PMC6870034

[65] Kamagata K, Motoi Y, Abe O, Shimoji K, Hori M, et al (2012) White matter alteration of the cingulum in Parkinson disease with and without dementia: evaluation by diffusion tensor tract-specific analysis. Am J Neuroradiology 33: 890–95. 10.3174/ajnr.A2860 PMC7968830 22241380

[66] Hughes AJ, Daniel SE, Ben Shlomo Y, Lees AJ (2000) The accuracy of diagnosis of parkinsonian syndromes in a specialist movement disorder service. Brain 125: 861–70. 10.1093/brain/awf080 11912118

[67] Jeurissen B, Leemans A, Tournier JD, Jones DK, Sijbers J (2012) Investigating the prevalence of complex fiber configurations in white matter tissue with diffusion magnetic resonance imaging. Hum Brain Mapp 19. doi: 10.1002/hbm.22099 10.1002/hbm.22099 PMC6870534 22611035

[68] Vos SB, Jones DK, Jeurissen B, Vierqever MA, Leemans A (2012) The influence of complex white matter architecture on the mean diffusivity in diffusion tensor MRI of the human brain. Neuroimage 59: 2208–16. 22005591 10.1016/j.neuroimage.2011.09.086 PMC7613439

[69] Descoteaux M, Deriche R, Knösche TR, Anwander A (2009) Deterministic and probabilistic tractography based on complex fibre orientation distributions. IEEE Trans Med Imaging 28: 269–86. 19188114 10.1109/TMI.2008.2004424

[70] Jeurissen B, Leemans A, Jones DK, Tournier JD, Sijbers J (2011) Probabilistic fiber tracking using the residual bootstrap with constrained spherical deconvolution. Hum Brain Mapp 32: 461–79. 21319270 10.1002/hbm.21032 PMC6869960

[71] Wedeen VJ, Wang RP, Schmahmann JD, Benner T, Tsenq WY, et al (2008) Diffusion spectrum magnetic resonance imaging (DSI) tractography of crossing fibers. Neuroimage 41: 1267–77. 18495497 10.1016/j.neuroimage.2008.03.036

[72] Hrabětová S, Nicholson C (2007) Biophysical Properties of Brain Extracellular Space Explored with Ion-Selective Microelectrodes, Integrative Optical Imaging and Related Techniques. In: Michael AC, Borland LM, editors. Electrochemical Methods for Neuroscience. Boca Raton (FL): CRC Press; Chapter 10. 21204394

[73] Douaud G, Jbabdi S, Behrens TE, Menke RA, Gass A, et al (2011) DTI measures in crossing-fibre areas: increased diffusion anisotropy reveals early white matter alteration in MCI and mild Alzheimer's disease. Neuroimage 55: 880–90. 21182970 10.1016/j.neuroimage.2010.12.008 PMC7116583

[74] Deprez S, Billiet T, Sunaert S, Leemans A (2013) Diffusion tensor MRI of chemotherapy-induced cognitive impairment in non-CNS cancer patients: a review. Brain Imaging Behav 18: In press. 10.1007/s11682-012-9220-1 23329357

